# Chemical Composition of Lipophilic Compounds From Rice (*Oryza sativa*) Straw: An Attractive Feedstock for Obtaining Valuable Phytochemicals

**DOI:** 10.3389/fpls.2022.868319

**Published:** 2022-03-22

**Authors:** Mario J. Rosado, Gisela Marques, Jorge Rencoret, Ana Gutiérrez, José C. del Río

**Affiliations:** Instituto de Recursos Naturales y Agrobiología de Sevilla, CSIC, Seville, Spain

**Keywords:** rice straw, phytochemicals, fatty acids, sterols, sterol glucosides, tocopherols

## Abstract

Rice (*Oryza sativa* L.) straw is a highly abundant, widely available, and low cost agricultural waste that can be used as a source to extract valuable phytochemicals of industrial interest. Hence, in the present work, the chemical composition of the lipophilic compounds present in rice straw was thoroughly characterized by gas chromatography and mass spectrometry using medium-length high-temperature capillary columns, which allowed the identification of a wide range of lipophilic compounds, from low molecular weight fatty acids to high molecular weight sterols esters, sterol glucosides, or triglycerides in the same chromatogram. The most abundant lipophilic compounds in rice straw were fatty acids, which accounted for up to 6,400 mg/kg (41.0% of all identified compounds), followed by free sterols (1,600 mg/kg; 10.2%), sterol glucosides (1,380 mg/kg; 8.8%), fatty alcohols (1,150 mg/kg; 7.4%), and triglycerides (1,140 mg/kg; 7.3%), along with lower amounts of high molecular weight wax esters (900 mg/kg; 5.8%), steroid ketones (900 mg/kg; 5.8%), monoglycerides (600 mg/kg; 3.8%), alkanes (400 mg/kg; 2.6%), diglycerides (380 mg/kg; 2.4%), sterol esters (380 mg/kg; 2.4%), tocopherols (340 mg/kg; 2.2%), and steroid hydrocarbons (60 mg/kg; 0.4%). This information is of great use for the valorization of rice straw to obtain valuable lipophilic compounds of interest for the nutraceutical, pharmaceutical, cosmetic, and chemical industries. Moreover, this knowledge is also useful for other industrial uses of rice straw, as in pulp and papermaking, since some lipophilic compounds are at the origin of the so-called pitch deposits during pulping.

## Introduction

Rice (*Oryza sativa* L.) is one of the most important staple food crops used for human nutrition worldwide. In 2020, paddy fields accounted for up to 164 million cultivated hectares with a global rice production of 757 million Mt., with Asia contributing 90% of the world rice production followed by Africa (5%), the Americas (4%), and Europe (1%; FAOSTAT, 2022). Rice harvesting generates large amounts of wastes such as rice straw, which includes stems, leaves, and spikelets. With an estimated grain/straw ratio of around 1.5 (Lal, 2005), the annual world production of rice straw is estimated to be around 1,130 million Mt. Nearly half of the rice straw is burnt for cogeneration of heat and power with the subsequent environmental problems while the rest is traditionally used as fodder or left for decomposition in landfills (Matsumura et al., 2005; Wang et al., 2016; Bhattacharyya et al., 2020; Kumar and Verma, 2021). Rice straw is also widely used as a raw material for papermaking in a variety of countries (Kaur et al., 2017; Elhelece, 2020). In recent years, however, there is great interest in developing approaches to exploit the potential of rice straw for biorefining, through conversion to biofuels and bioproducts (Abraham et al., 2016).

Rice straw is a lignocellulosic material essentially constituted of cellulose (24.0%), hemicelluloses (27.8%), and lignin (13.5%), with important amounts of ash (17%) that correspond mostly to silica (Rosado et al., 2021). Due to the significant amounts of carbohydrates and lignin, and to its low price and high availability, rice straw has been considered a suitable feedstock for the production of biofuels, biochemicals, and biobased materials in the context of lignocellulosic biorefineries (Lal, 2005; Lu and Hsieh, 2012; Kalita et al., 2015; Abraham et al., 2016; Kumar et al., 2016; Gou et al., 2018; Swain et al., 2019; Bhattacharyya et al., 2020; Sharma et al., 2020). In addition, rice straw also presents significant amounts of lipophilic compounds (3.4%) that can also be used to obtain valuable phytochemicals of industrial interest (Rosado et al., 2021). Most of the functionally important plant-based compounds derive from food processing as by-products. Nevertheless, lignocellulosic wastes (cereal by-products such as wheat straw, rice husks, or maize fibers, among others) that are easily available at a very low cost are considered alternative sources for obtaining valuable phytochemicals (del Río et al., 2013a,b, 2015; Marques et al., 2020).

Plant lipophilic compounds comprise a wide range of chemical products (e.g., hydrocarbons, fatty acids, fatty alcohols, aldehydes, acylglycerols, terpenoids, and steroids) that present many applications in the pharmaceutical, nutraceutical, cosmetic, food, or chemical industries (Hernandez, 2005; Metzger and Bornscheuer, 2006; Tao, 2007; Rombaut et al., 2014; Sin et al., 2014; Carciochi et al., 2017; Attard et al., 2018). For example, fatty acids and acylglycerols are widely used for the production of biodiesel (Zhang et al., 2013) as well as cosmetics (Kalustian, 1985). Among the fatty acids, linoleic acid is an omega-6 essential unsaturated fatty acid of interest for the food and nutraceutical industries. Moreover, linoleic acid is also used in pharmaceutical and cosmetic products and is considered to influence the metabolic processes in the skin and to promote the activity of vitamins A and E and the restoration of the barrier properties of stratum corneum (Huang et al., 1999). On the other hand, plant sterols are also of interest in the food and nutraceutical industry because, when used as functional ingredients in foods, contribute to lowering blood cholesterol levels (Wilson et al., 2000; Quílez et al., 2003; Jones and AbuMweis, 2009), play an important role in the regulation of cardiovascular disease, and exhibit anticancer properties (Iwatsuki et al., 2003; Jones and AbuMweis, 2009). In addition, sterol ferulates, which are widely present in cereal bran oils, present anti-inflammatory effects and are also effective antitumor-promoters (Akihisa et al., 2000). Tocopherols and tocotrienols also have antioxidant properties and play a role in the prevention of certain types of cancer, heart disease, and other chronic ailments (Shahidi and De Camargo, 2016).

On the other hand, however, the presence of lipophilic compounds in the raw material can be detrimental during some industrial processing operations, such as during pulp and paper manufacturing. The lipophilic compounds can form organic deposits (called pitch) during pulp and paper production that have negative effects in the product as well as in the machinery and resulting in significant economic losses (del Río et al., 1998, 2000; Back and Allen, 2000; Gutiérrez et al., 2001, 2004). During alkaline cooking, the different lipophilic compounds exhibit different behavior. Thus, acylglycerols are completely hydrolyzed, and fatty acids are extensively dissolved forming fatty acid soaps. However, neutral compounds, particularly free sterols, fatty alcohols, sterol glycosides, steroid hydrocarbons, and steroid ketones, survive cooking and are difficult to remove in the washing stages due to their low water solubility, and therefore can be at the origin of the sticky deposits (pitch) in the pulp and in the machinery. It is, therefore, essential to know the exact nature of the lipophilic compounds in rice straw in order to maximize the exploitation of this important agricultural waste.

However, despite the importance and the enormous quantities of rice straw produced annually, there is a lack of studies reporting the detailed chemical composition of the lipophilic compounds in this material. Only a few studies describing the chemical composition of the lipophilic compounds in rice straw have been published, but with limited success. A previous work (Xiao et al., 2001) examined the composition of the lipophilic compounds in rice straw and reported free fatty acids, sterols, waxes, sterol esters, and triglycerides; however, the occurrence of significant amounts of resin acids, a group of compounds that is exclusively restricted to conifers, as well as other compounds such as cholesterol, which is not a typical plant sterol, raised the question of whether the sample studied was contaminated or whether the lipophilic compounds were properly identified. Another study reported only the composition of free fatty acids in rice straw, which were dominated by palmitic, and oleic acids, with significant amounts of linoleic and stearic acids (Zemnukhova et al., 2015). Finally, Zhao et al. (2007) also reported some lipophilic compounds in rice straw, including some tocopherols and steroid compounds, although the identities of the reported steroids presented many inconsistencies and most of them may have been misidentified because they are rarely present in plants. Therefore, a comprehensive and detailed description of the chemical composition of the lipophilic compounds present in rice straw is still pending.

In this work, the lipophilic compounds in rice straw were analyzed by gas chromatography-mass spectrometry (GC-MS) using a medium-length, high-temperature capillary column, with thin films, according to the method developed by our group that allowed the elution and identification of a wide range of components, from low molecular weight fatty acids to high molecular weight sterol esters or triglycerides (Gutiérrez et al., 1998, 2004). The results presented here will greatly improve our understanding of the lipophilic compounds in rice straw and will help to maximize the exploitation of this important agricultural waste.

## Materials and Methods

### Samples

Rice (*Oryza sativa* L., var. Indica, Puntal) was harvested from a paddy field in Isla Mayor (Southern Spain). Samples of rice straw were brought to the laboratory, air-dried, and crushed using an IKA knife mill (Janke and Kunkel, Staufen, Germany) with 1-mm screen. The samples were then extracted with acetone in a Soxhlet for 8 h. The extracts were brought to dryness using a rotary evaporator and were further determined gravimetrically, accounting for 3.4% ± 0.1 (dry-basis). The determination was performed in triplicate. The moisture content of the rice straw was determined by drying the sample in an oven at 105°C for 24 h.

### GC-MS Analysis of Lipophilic Compounds in Rice Straw

The acetone extracts were redissolved in chloroform and subsequently analyzed by GC-MS both underivatized, and after derivatization with bis(trimethylsilyl)trifluoroacetamide (BSTFA), using the equipment and experimental conditions previously described (del Río et al., 2016). The different compounds were identified by comparison of their mass spectra with those in the NIST library, by comparison with literature (Lauer et al., 1970; Curstedt, 1974; Evershed et al., 1989; Snyder et al., 1993; Gutiérrez and del Río, 2001; del Río et al., 2009, 2013a, 2015, 2016), and when possible by comparison with authentic standards (alkanes from *n*-octadecane to *n*-hentriacontane, alcohols from *n*-hexadecanol to *n*-octacosanol, saturated fatty acids from *n*-tetracosanoic acid to *n*-eicosanoic acid, the unsaturated fatty acids oleic, linoleic, and linolenic acids; the sterols campesterol, stigmasterol, sitosterol, and their respective 3β-d-glucopyranosides; the sterol esters cholesteryl palmitate, cholesteryl oleate, and cholesteryl linoleate; the wax ester tetradecyl tetradecanoate; and the acylglycerols 1-monopalmitin, 1,3-dipalmitin, tripalmitin, triolein, and trilinolein). Quantification was performed by using a mixture of authentic external standards (palmitic acid, linoleic acid, stigmasterol, sitosterol, cholesteryl linoleate, sitosteryl 3β-d-glucopyranoside, 1-monopalmitin, 1,3-dipalmitin, tripalmitin, and tetracosane) in a concentration range between 0.1 and 1 mg/mL, and the calibration curves and response factors were determined for each of them. The correlation coefficient was higher than 0.99 in all cases. All peaks were quantified by peak area. Quantification was given as the mean of three replicates.

## Results and Discussion

### Lipophilic Constituents in Rice Straw

**Figure 1:**
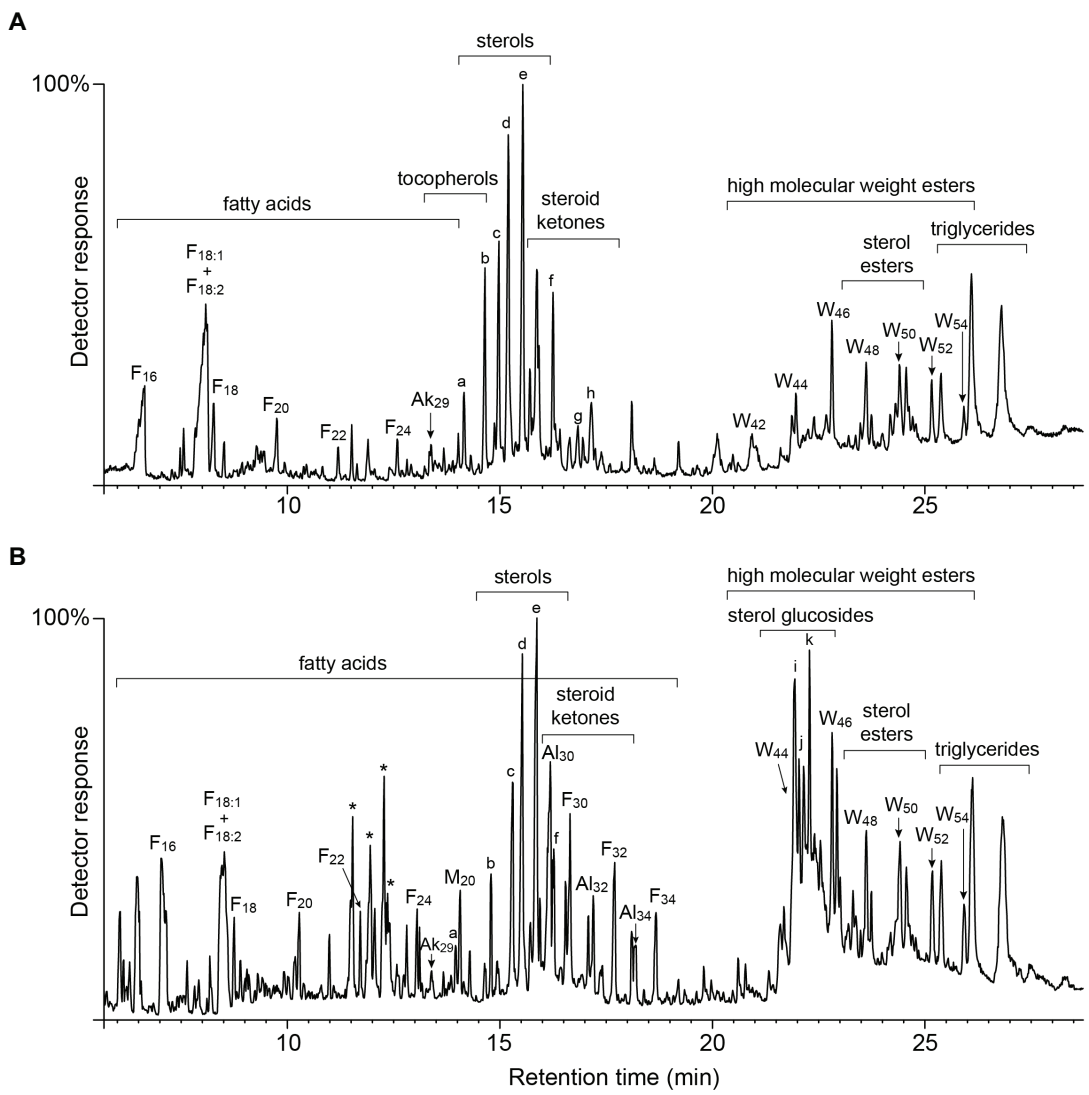
GC-MS chromatograms of the acetone extracts from rice straw, **(A)** underivatized, and **(B)** as TMS-ether derivatives. F(n), *n*-fatty acid series; Al(n), *n*-fatty alcohol series; Ak(n), *n*-alkanes; M(n), monoglycerides; and W(n), high molecular weight esters. Labels for selected compounds are as: (**a**), γ-tocopherol; (**b**), α-tocopherol; (**c**), campesterol; (**d**), stigmasterol; (**e**), sitosterol; (**f**), stigmast-4-en-3-one; (**g**), ergostane-3,6-dione; (**h**), stigmastane-3,6-dione; (**i**), campesteryl 3β-d-glucopyranoside; (**j**), stigmasteryl 3β-d-glucopyranoside; and (**k**), sistosteryl 3β-d-glucopyranoside. ^*^unknown compounds, possibly glycolipids, referred in the text.

**Figure 2:**
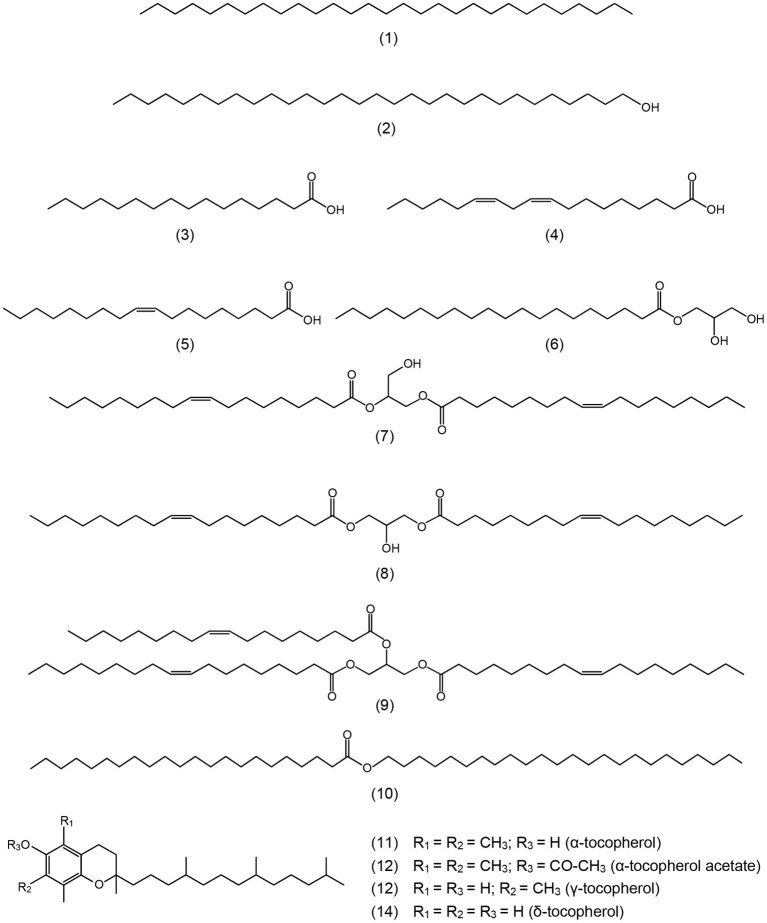
Structures representative of the main aliphatic compounds identified in the acetone extracts of rice straw and referred in the text. **1**: *n*-nonacosane; **2**: *n*-triacontanol; **3**: *n*-hexadecanoic (palmitic) acid; **4**: *cis*,*cis*-octadeca-9,12-dienoic (linoleic) acid; **5**: *cis*-octadec-9-enoic (oleic) acid; **6**: 2,3-dihydroxypropyl eicosanoate; **7**: 1,2-diolein; **8**: 1,3-diolein; **9**: triolein; **10**: docosanoic acid, tetracosyl ester; **11**: α-tocopherol; **12**: α-tocopherol acetate; **13**: γ-tocopherol; and **14**: δ-tocopherol.

**Figure 3:**
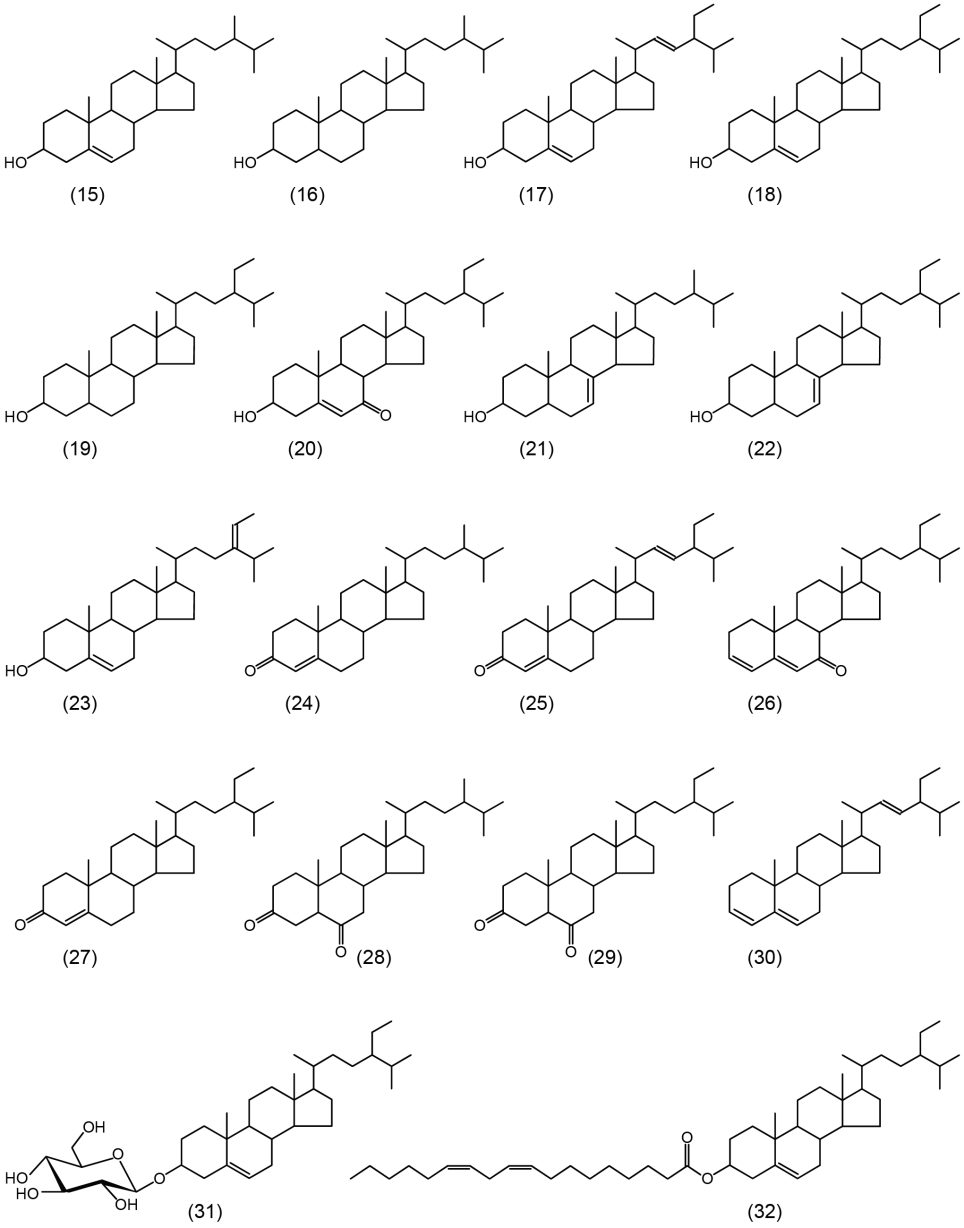
Structures of the main steroid compounds identified in the acetone extracts of rice straw and referred in the text. **15**: campesterol; **16**: campestanol; **17**: stigmasterol; **18**: sitosterol; **19**: stigmastanol; **20**: 7-oxo-sitosterol; **21**: Δ^7^-campesterol; **22**: Δ^7^-stigmastenol; **23**: Δ^5^-avenasterol; **24**: ergost-4-en-3-one; **25**: stigmasta-4,22-dien-3-one; **26**: stigmasta-3,5-dien-7-one; **27**: stigmast-4-en-3-one; **28**: ergostane-3,6-dione; **29**: stigmastane-3,6-dione; **30**: stigmasta-3,5,22-triene; **31**: sitosteryl 3β-d-glucopyranoside; and **32**: sitosteryl linoleate.

The lipophilic compounds in rice straw accounted for 3.4% on a dry-basis. The composition of the lipophilic extracts (both underivatized and as their TMS-ether derivatives) were analyzed by GC-MS using high-temperature medium-length capillary columns according to the method previously described, that allowed the identification of high molecular weight compounds, as sterol glycosides, sterol esters, high molecular weight wax esters, and triglycerides (Gutiérrez et al., 1998, 2004). The chromatograms of the underivatized and the TMS-ether derivatives of the lipophilic compounds from rice straw are shown in Figure 1. The identities and abundances (as mg/kg, dry-weight basis) of the main lipophilic compounds identified are detailed in Table 1. Several classes of compounds were identified by GC-MS, including *n*-fatty acids, *n*-alkanes, tocopherols, steroid hydrocarbons, steroid ketones, free sterols, sterol esters, sterol glucosides, mono-, di-, and triglycerides, and high molecular weight ester waxes. Structures representatives of the main aliphatic compounds identified in rice straw are depicted in Figure 2, whereas structures representatives of the main steroid compounds are shown in Figure 3.

**Table 1:** Composition and abundance (mg/kg, on a dry-basis) of the compounds identified in rice straw (in parenthesis are the percentages referred to the total compounds identified).

**Compounds**	**mg/kg (%)**		**mg/kg (%)**
***n*-Alkanes**	**400 ± 20 (2.6%)**	**Triglycerides**	**1,140 ± 120 (7.3%)**
*n*-pentacosane	32	C_51_ (tripalmitin)	180
*n*-hexacosane	12	C_55_ (palmitoyldiolein + palmitoyldilinolein)	492
*n*-heptacosane	70	C_57_ (trilinolein + triolein, **9**)	468
*n*-octacosane	30		
*n*-nonacosane (**1**)	152	**High molecular weight esters**	**900 ± 50 (5.8%)**
*n*-triacontane	40	esters C_42_	36
*n*-hentriacontane	64	esters C_44_	116
		esters C_46_ (**10**)	204
***n*-Fatty alcohols**	**1,150 ± 30 (7.4%)**	esters C_48_	180
*n*-octacosanol	102	esters C_50_	164
*n*-triacontanol (**2**)	440	esters C_52_	124
*n*-dotriacontanol	370	esters C_54_	76
*n*-tetratriacontanol	176		
*n*-hexatriacontanol	62	**Tocopherols**	**340 ± 40 (2.2%)**
		α-tocopherol (**11**)	220
***n*-Fatty acids**	**6,400 ± 200 (41.0%)**	α-tocopherol acetate (**12**)	30
*n*-hexadecanoic acid (**3**)	650	γ-tocopherol (**13**)	80
*n*-heptadecanoic acid	110	δ-tocopherol (**14**)	10
C_18:2_ (*cis,cis*-octadeca-9,12-dienoic acid, **4**)	1,200		
C_18:1_ (*cis*-octadec-9-enoic acid, **5**)	740	**Sterols**	**1,600 ± 50 (10.2%)**
*n*-octadecanoic acid	394	campesterol (**15**)	312
*n*-nonadecanoic acid	80	campestanol (**16**)	10
*n*-eicosanoic acid	375	stigmasterol (**17**)	528
*n*-heneicosanoic acid	100	sitosterol (**18**)	600
*n*-docosanoic acid	286	stigmastanol (**19**)	80
*n*-tricosanoic acid	114	7-oxo-sitosterol (**20**)	14
*n*-tetracosanoic acid	336	Δ^7^-campesterol (**21**)	36
*n*-pentacosanoic acid	108	Δ^7^-stigmastenol (**22**)	10
*n*-hexacosanoic acid	165	Δ^5^-avenasterol (**23**)	10
*n*-heptacosanoic acid	40		
*n*-octacosanoic acid	296	**Steroid ketones**	**900 ± 20 (5.8%)**
*n*-nonacosanoic acid	54	ergost-4-en-3-one (**24**)	112
*n*-triacontanoic acid	520	stigmasta-4,22-dien-3-one (**25**)	360
*n*-hentriacontanoic acid	80	stigmasta-3,5-dien-7-one (**26**)	30
*n*-dotriacontanoic acid	464	stigmast-4-en-3-one (**27**)	248
*n*-tritriacontanoic acid	50	ergostane-3,6-dione (**28**)	50
*n*-tetratriacontanoic acid	238	stigmastane-3,6-dione (**29**)	100
**Monoglycerides**	**600 ± 80 (3.8%)**	**Steroid hydrocarbons**	**60 ± 10 (0.4)**
2,3-dihydroxypropyl hexadecanoate	68	stigmasta-3,5-diene	20
2,3-dihydroxypropyl octadeca-9,12-dienoate	86	stigmasta-3,5,22-triene (**30**)	40
2,3-dihydroxypropyl octadec-9-enoate	96		
2,3-dihydroxypropyl octadecanoate	40	**Sterol glucosides**	**1,380 ± 150 (8.8%)**
2,3-dihydroxypropyl eicosanoate (**6**)	220	campesteryl 3β-d-glucopyranoside	370
2,3-dihydroxypropyl docosanoate	56	stigmasteryl 3β-d-glucopyranoside	310
2,3-dihydroxypropyltetracosanoate	34	sitosteryl 3β-d-glucopyranoside (**31**)	700
**Diglycerides**	**380 ± 50 (2.4%)**	**Sterol sters**	**380 ± 30 (2.4%)**
1,2-dipalmitin	20	campesterol palmitate	26
1,3-dipalmitin	16	campesterol oleate/campesterol linoleate	58
1,2-palmitoylolein+1,2-palmitoyllinolein	60	stigmasterol palmitate	32
1,3-palmitoylolein+1,3-palmitoyllinolein	56	stigmasterol oleate/stigmasterol linoleate	76
1,2-diolein (**7**) +1,2-dilinolein+1,2-oleyllinolein	144	sitosterol palmitate	50
1,3-diolein (**8**) +1,3-dilinolein+1,3-oleyllinolein	84	sitosterol oleate/sitosterol linoleate (**32**)	138

Bold numbers in parenthesis refer to the structures depicted in Figures 2, 3.

**Figure 4:**
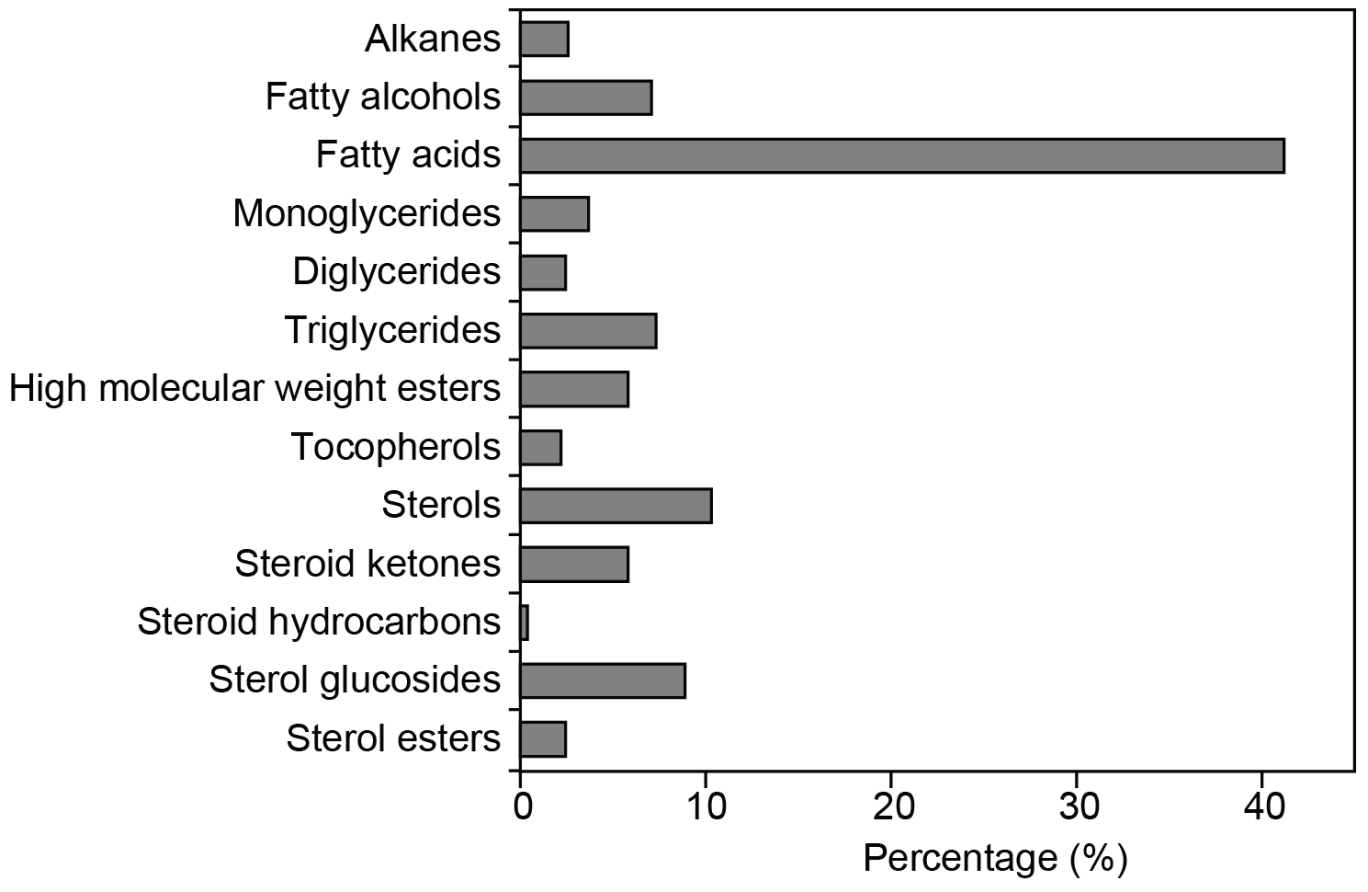
Percentage of the main classes of lipophilic compounds identified in the acetone extracts of rice straw.

The relative abundances of the different classes of lipophilic compounds in rice straw are depicted in the histogram of Figure 4. The most abundant class of lipophilic compounds were series of fatty acids, that accounted for up to 41.0% of all compounds identified, followed by free sterols (10.2%), sterol glucosides (8.8%), fatty alcohols (7.4%), and triglycerides (7.3%), together with lower amounts of high molecular weight wax esters (5.8%), steroid ketones (5.8%), monoglycerides (3.8%), alkanes (2.6%), diglycerides (2.4%), sterol esters (2.4%), tocopherols (2.2%), and steroid hydrocarbons (0.4%). A series of peaks appeared in the chromatogram of the TMS-ether derivatives around 11–13 min (marked with asterisks), but their identities could not be fully established. The mass spectra of these peaks exhibited characteristic fragments of carbohydrates (*m/z* 147, 217, 361), suggesting that they might belong to glycolipids, but it did not show any other diagnostic fragment that might provide additional clues to their identities. Some glycolipids have been reported to occur among the lipophilic compounds of rice extracts, as the mono- and digalactosyl monoacylglycerols and the mono- and digalatosyl diacylglycerols (Moazzami et al., 2011). Interestingly, these peaks from unknown glycolipids also appeared in rice husks (Marques et al., 2020), suggesting that they might be typical compounds of rice.

It is important to note that the acetone extracts in rice straw represent 3.4% (dry-basis) or 34 mg/kg, while the sum of the amounts of the different lipophilic compounds identified in Table 1 is around 15.6 mg/kg. This discrepancy is most likely due to the occurrence in the acetone extracts of polar compounds that have not been quantified, as well as high molecular weight compounds that are out of the analytical window of our procedure.

### Aliphatic Series

**Figure 5:**
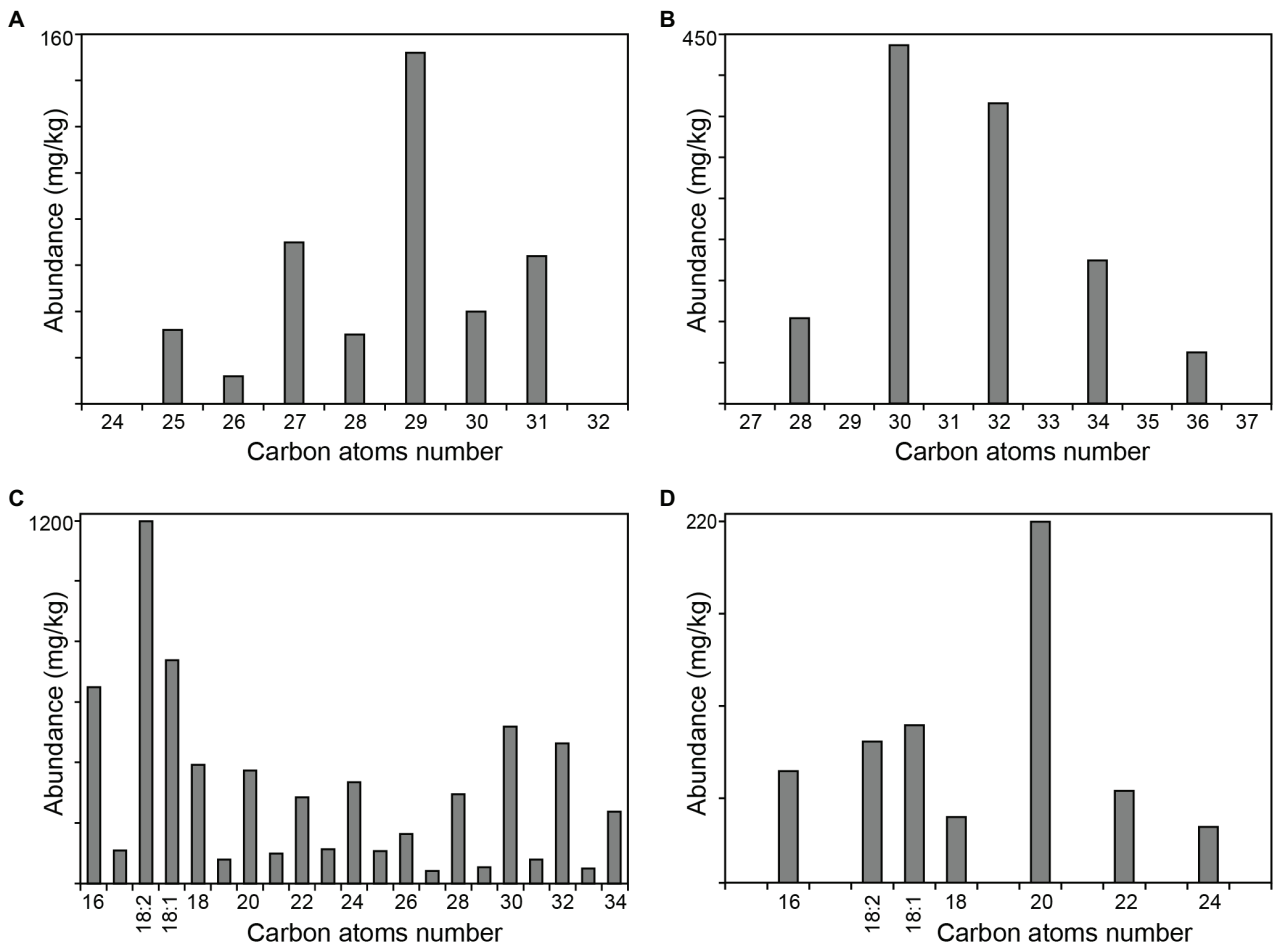
Distribution of the main aliphatic series identified in the extracts of rice straw. **(A)** series of *n*-alkanes; **(B)** series of *n*-fatty alcohols; **(C)** series of *n*-fatty acids; and **(D)** series of monoglycerides. The histograms are scaled up to the abundance of the major compound in the series.

The main aliphatic compounds identified in rice straw were series of free *n*-fatty acids, acylglycerols (mono-, di-, and triglycerides), high molecular weight esters, *n*-fatty alcohols, *n*-alkanes, as well as small amounts of tocopherols. The distributions of the series of *n*-alkanes, *n*-fatty alcohols, *n*-fatty acids, and monoglycerides are represented in the histograms of Figure 5.

The series of *n*-alkanes in rice straw accounted for 400 mg/kg, and ranged from *n*-pentacosane (C_25_) to *n*-hentriacontane (C_31_), with a strong predominance of the odd carbon atom number homologues and with maximum for *n*-nonacosane (C_29_; **1**), that accounted for 152 mg/kg, as shown in Figure 5A. This is the first time that the series of *n*-alkanes have been reported in rice straw. A previous paper indicated the occurrence of tetratriacontane (C_34_) and pentatriacontane (C_35_) in rice straw (Zhao et al., 2007); however, the identities of these alkanes may have been erroneously assigned as their relative retention times did not correspond to these compounds; and in our opinion, this paper presented many inconsistencies and most of the identifications reported there should be taken with caution. Alkanes could not be identified in the respective rice husks waste (Marques et al., 2020) but were reported in similar amounts (371 m/kg) in the related wheat straw (del Río et al., 2013a).

The series of fatty alcohols was also identified in rice straw in important amounts, accounting for a total of 1,150 mg/kg. The series ranged from *n*-octacosanol (C_28_) to *n*-hexatriacontanol (C_36_), with the exclusive presence of the even carbon atom number homologues, and with maximum for *n*-triacontanol (C_30_; **2**) that accounted for 440 mg/kg, as depicted in Figure 5B. This is the first time that the series of fatty alcohols were reported among the lipophilic compounds of rice straw as they were not identified in previous studies (Xiao et al., 2001; Zhao et al., 2007). Fatty alcohols were also reported, although to a lesser extent (340 mg/kg) in the respective rice husks waste (Marques et al., 2020), but higher amounts of alcohols (1,615 mg/kg) were reported in the related wheat straw (del Río et al., 2013a).

Free fatty acids were the most abundant class of lipophilic compounds in rice straw, accounting for a total of 6,400 mg/kg. The content of fatty acids was higher than in the respective rice husks, with 2,770 mg/kg (Marques et al., 2020), and in the related wheat straw, with only 2,080 mg/kg (del Río et al., 2013a). The series of saturated fatty acids ranged from *n*-hexadecanoic acid (C_16_; palmitic acid, **3**) to *n*-tetratriacontanoic acid (C_34_), with a strong predominance of the even carbon atom number homologues, and a bimodal distribution with two maxima for *n*-hexadecanoic acid (650 mg/kg) and *n*-triacontanoic acid (C_30_; 520 mg/kg), as depicted in Figure 5C. Large amounts of mono- (C_18:1_; 740 mg/kg) and diunsaturated (C_18:2_; 1,200 mg/kg) fatty acids were also identified. Although the experimental conditions used here, particularly the GC column, do not allow their unequivocal identification, they most likely correspond to oleic (C_18:1_) and linoleic (C_18:2_) acids, the most common unsaturated fatty acids in similar lignocellulosic residues. The distribution of free fatty acids is similar to that found in rice husks (Marques et al., 2020). Previous works (Xiao et al., 2001; Zemnukhova et al., 2015) also reported a similar distribution of fatty acids in rice straw but failed to detect the high molecular weight fatty acids above tetracosanoic acid (C_24_), which amounted up to a third of the total fatty acids identified. In the present work, the identification of the high molecular weight fatty acids was possible by the use of the methodology developed in our laboratories that used medium-length high-temperature capillary columns (Gutiérrez et al., 1998). Surprisingly, a previous work also reported the occurrence of abietic acid in rice straw (Xiao et al., 2001). However, abietic acid, like all other resin acids, is exclusively restricted to conifers and cannot occur in rice (a monocotyledonous plant), raising the question of whether this compound was misidentified or whether the sample studied was contaminated.

Acylglycerols (including mono-, di-, and triglycerides) were present in important amounts in rice straw, accounting for a total of 2,120 mg/kg. Triglycerides were the most abundant acylglycerols, accounting for 1,140 mg/kg, followed by monoglycerides (600 mg/kg) and diglycerides (380 mg/kg). In any case, the content of triglycerides in rice straw was much lower than those present in the respective rice husks where they accounted for up to 10,400 mg/kg (Marques et al., 2020). Previous papers only reported the occurrence of di- and triglycerides in rice straw but failed to detect monoglycerides (Xiao et al., 2001). In the present work, the series of monoglycerides were identified in the range from 2,3-dihydroxypropyl hexadecanoate (C_16_, 1-monopalmitin) to 2,3-dihydroxypropyl tetracosanoate (C_24_), with the occurrence of only the even carbon atom number homologues, and with 2,3-dihydroxypropyl eicosanoate (C_20_, **6**) being the most abundant one (220 mg/kg), as depicted in Figure 5D. Significant amounts of the unsaturated 2,3-dihydroxypropyl octadec-9,12-dienoate (C_18:2_, 1-monolinolein; 86 mg/kg) and 2,3-dihydroxypropyl octadec-9-enoate (C_18:1_, 1-monoolein; 96 mg/kg) were also identified. On the other hand, the diglycerides identified in rice straw corresponded to different combinations of the saturated palmitic acid (C_16_) and the unsaturated linoleic (C_18:2_) and oleic (C_18:1_) acids, with different substitution patterns (forming 1,2- and 1,3-isomers). The individual diglycerides could be identified by their mass spectra, as previously published (Curstedt, 1974). The diglycerides identified in rice straw were 1,2- and 1,3-dipalmitin, 1,2- and 1,3-palmitoylolein, 1,2- and 1,3-palmitoyllinolein, 1,2- and 1,3-diolein (**7**, and **8**), 1,2- and 1,3-dilinolein, and 1,2- and 1,3-oleyllinolein. Among the different diglycerides, a predominance of the 1,2- over the 1,3-isomers was observed, as also occurred with the diglycerides identified in the respective rice husks (Marques et al., 2020). A previous work reported only the occurrence of “dipalmitin” but did not indicate the substitution pattern (Xiao et al., 2001). Finally, triglycerides in rice straw appeared as a mixture of several compounds that eluted in three main chromatographic peaks that were separated by total carbon number (C_51_, C_55_, and C_57_); however, identification of individual triglycerides in each peak could be achieved based on their mass spectra, as already reported (Lauer et al., 1970; del Río et al., 2016). The analyses indicated that the main triglycerides in rice straw were tripalmitin (180 mg/kg), palmitoyldiolein/palmitoyllinolein (492 mg/kg), and trilinolein/triolein (**9**, 468 mg/kg); a similar distribution pattern has been previously reported (Xiao et al., 2001).

A series of high molecular weight ester waxes were also found in rice straw in significant amounts (900 mg/kg). This series was formed by combinations of different long-chain fatty acids with different long-chain fatty alcohols that produced a wide variety of various long-chain ester waxes in the range from C_42_ to C_54_. The identities of the different individual high molecular weight ester waxes were determined from their mass spectra, as already reported (del Río et al., 2009, 2013a). The mass spectra of long-chain esters were characterized by a base peak that corresponded to the protonated acid ion. Hence, the base peak provides information about the acid moiety while the molecular ion provided information of the total number of carbon atoms in the ester. It was then possible to determine the contribution of individual esters in every chromatographic peak by mass spectrometric determination of the molecular ion and the base peak. Quantitation of individual esters was accomplished by integrating the areas in the chromatographic profiles of the ions characteristic for the acidic moiety. The detailed composition of the different high molecular weight ester waxes identified in rice straw is shown in Table 2. The esterified fatty acids ranged from hexadecanoic (C_16_) to tetracosanoic acid (C_24_), whereas the esterified alcohols ranged from eicosanol (C_20_) to tetratriacontanol (C_34_). It is important to note the absence of unsaturated fatty acids forming long-chain ester waxes, despite the high abundance of unsaturated fatty acids (oleic and linoleic acids) present in free form in rice straw. The most prominent high molecular weight esters in rice straw were C_46_ (204 mg/kg), which was primarily constituted by docosanoic acid, tetracosyl ester (**10**), that accounted from 92 mg/kg. The high molecular weight ester waxes identified in the present work have not been reported previously in rice straw, although the occurrence of palmitic acid, palmityl ester, a C_32_ wax ester, along with smaller amounts of unsaturated esters, was reported in a previous paper (Xiao et al., 2001), but they could not be detected in the present work. High molecular weight esters were detected only in low amounts (60 mg/kg) in the respective rice husks (Marques et al., 2020), and in similar amounts (915 mg/kg) in the related wheat straw (del Río et al., 2013a).

**Table 2:** Composition and abundance (mg/kg, on a dry-basis) of the different individual esters found among the waxes identified in the extracts of rice straw.

Compound	Fatty acid:Fatty alcohol	Abundance
**Esters C**_**42**_		**36**
hexadecanoic acid, hexacosyl ester	C_16_:C_26_	10
octadecanoic acid, tetracosyl ester	C_18_:C_24_	6
eicosanoic acid, docosyl ester	C_20_:C_22_	10
docosanoic acid, eicosyl ester	C_22_:C_20_	10
**Esters C**_**44**_		**116**
hexadecanoic acid, octacosyl ester	C_16_:C_28_	16
octadecanoic acid, hexacosyl ester	C_18_:C_26_	10
eicosanoic acid, tetracosyl ester	C_20_:C_24_	40
docosanoic acid, docosyl ester	C_22_:C_22_	50
**Esters C**_**46**_		**204**
hexadecanoic acid, triacontyl ester	C_16_:C_30_	50
octadecanoic acid, octacosyl ester	C_18_:C_28_	12
eicosanoic acid, hexacosyl ester	C_20_:C_26_	30
docosanoic acid, tetracosyl ester (**10**)	C_22_:C_24_	92
tetracosanoic acid, docosyl ester	C_24_:C_22_	20
**Esters C**_**48**_		**180**
hexadecanoic acid, dotriacontyl ester	C_16_:C_32_	42
octadecanoic acid, triacontyl ester	C_18_:C_30_	30
eicosanoic acid, octacosyl ester	C_20_:C_28_	30
docosanoic acid, hexacosyl ester	C_22_:C_26_	32
tetracosanoic acid, tetracosyl ester	C_24_:C_24_	46
**Esters C**_**50**_		**164**
hexadecanoic acid, tetratriacontyl ester	C_16_:C_34_	20
octadecanoic acid, dotriacontyl ester	C_18_:C_32_	16
eicosanoic acid, triacontyl ester	C_20_:C_30_	56
docosanoic acid, octacosyl ester	C_22_:C_28_	42
tetracosanoic acid, hexacosyl ester	C_24_:C_26_	30
**Esters C**_**52**_		**124**
eicosanoic acid, dotriacontyl ester	C_20_:C_32_	36
docosanoic acid, triacontyl ester	C_22_:C_30_	62
tetracosanoic acid, octacosyl ester	C_24_:C_28_	26
**Esters C**_**54**_		**76**
eicosanoic acid, tetratriacontyl ester	C_20_:C_34_	20
docosanoic acid, dotriacontyl ester	C_22_:C_32_	28
tetracosanoic acid, triacontyl ester	C_24_:C_30_	28

Bold number in parenthesis refers to the structure depicted in Figure 2.

Finally, different tocopherols were also found among the lipophilic compounds of rice straw, accounting for a total of 340 mg/kg. Tocopherols were identified by comparison with the mass spectra previously published (Snyder et al., 1993; del Río et al., 2015). The most predominant tocopherol was α-tocopherol (**11**) that accounted for 220 mg/kg, followed by γ-tocopherol (**13**, 80 mg/kg) and minor amounts of α-tocopherol acetate (**12**, 30 mg/kg) and δ-tocopherol (**14**, 10 mg/kg). The occurrence of α-tocopherol and α-tocopherol acetate in rice straw was already reported in a previous paper, although it failed to detect γ- and δ-tocopherols (Zhao et al., 2007). Among the tocopherols, α-tocopherol is the most abundant and active form of vitamin E in nature. Tocopherols have antioxidant properties and have a role in the prevention of certain types of cancer, as well as heart and other diseases (Shahidi and De Camargo, 2016). Tocopherols have been reported in rice husks (40 mg/kg), but in lower amounts than those found in the respective rice straw (Marques et al., 2020).

### Steroid Compounds

Different families of steroid compounds were identified among the lipophilic compounds of rice straw, including steroid ketones, steroid hydrocarbons, free sterols, sterol glucosides, and sterol esters (Table 1; Figure 3), that in total amounted up to 4,320 mg/kg.

Free sterols were the most predominant class of steroids in rice straw, accounting for 1,600 mg/kg. The most important sterols identified were campesterol (**15**, 312 mg/kg), stigmasterol (**17**, 528 mg/kg), sitosterol (**18**, 600 mg/kg), and stigmastanol (**19**, 80 mg/kg). Other sterols were also identified in lower amounts, including campestanol (**16**, 10 mg/kg), 7-oxo-sitosterol (**20**, 14 mg/kg), Δ^7^-campesterol (**21**, 36 mg/kg), Δ^7^-stigmastenol (**22**, 10 mg/kg), and Δ^5^-avenasterol (**23**, 10 mg/kg). Only stigmasterol and sitosterol, together with minor amounts of cholesterol, which is not a typical plant sterol, were previously reported in rice straw (Xiao et al., 2001). However, a different paper (Zhao et al., 2007) reported a variety of rare and weird sterols (i.e., γ-sitosterol and stigmasta-3-en-6-ol, among others), but as said above, this paper presented many inconsistencies such as the identification of stigmasterol and 5,22-dien-3-stigmasterol (which are synonyms for the same compound) as two different sterols in the same sample, but could not detect sitosterol that is the most abundant sterol in rice straw (Zhao et al., 2007). Sterols were also found, although in lower amounts (250 mg/kg), in the respective rice husks (Marques et al., 2020). Likewise, lower amounts of sterols (1,121 mg/kg) were reported in the related wheat straw (del Río et al., 2013a).

Sterol glucosides were also found in rice straw in important amounts, accounting for 1,380 mg/kg. This is the first time that sterol glucosides have been reported in rice straw as they were not identified in previous studies (Xiao et al., 2001; Zhao et al., 2007). Their identification was made by comparison with the retention time and mass spectra of authentic standards (as their TMS-ether derivatives), as previously reported (Gutiérrez and del Río, 2001). The most abundant sterol glucoside in rice straw was sitosteryl 3β-d-glucopyranoside (**31**) that amounted up to 700 mg/kg, followed by campesteryl-3β-d-glucopyranoside (370 mg/kg) and stigmasteryl-3β-d-glucopyranoside (310 mg/kg). A similar distribution of sterol glucosides was found, although in much lower amounts (70 mg/kg) in the respective rice husks waste (Marques et al., 2020). Important amounts of sterol glucosides (680 mg/kg) were also reported in the related wheat straw (del Río et al., 2013a).

Significant amounts of sterol esters were also found in rice straw, accounting for 380 mg/g. Sterol esters were identified by comparison with the mass spectra of authentic standards that matched those previously published (Evershed et al., 1989), as well as by comparison with retention times and mass spectra of an enriched fraction of sterol esters isolated from abaca (del Río and Gutiérrez, 2006). The sterol esters identified corresponded to campesterol, stigmasterol, and sitosterol esterified with different fatty acids, particularly with palmitic, and with the mono- (C_18:1_) and diunsaturated (C_18:2_) fatty acids (that most likely correspond to oleic and linoleic acids). The most important sterol esters present in rice straw were a mixture of sitosterol esterified with the C_18:1_ and C_18:2_ unsaturated fatty acids, that most likely correspond to sitosterol oleate/sitosterol linoleate (**32**), accounting for 138 mg/kg. Small amounts of sterol esters (70 mg/kg) were found in the respective rice husks (Marques et al., 2020).

Steroid ketones were found in significant amounts in rice straw, accounting for a total of 900 mg/kg. The most important steroid ketones identified were ergost-4-en-3-one (**24**, 112 mg/kg), stigmasta-4,22-dien-3-one (**25**, 360 mg/kg), stigmast-4-en-3-one (**27**, 248 mg/kg), and stigmastane-3,6-dione (**29**, 100 mg/kg), together with minor amounts of stigmasta-3,5-dien-7-one (**26**, 30 mg/kg) and ergostane-3,6-dione (**28**, 50 mg/kg). A previous paper (Zhao et al., 2007) reported the occurrence of stigmast-4-en-3-one, although they also reported other steroid ketones, such as spinasterone, 22,23-dihydrospinasterone, and cholest-4-en-3-one-26-oic acid, which are rare and uncommon steroid ketones, which could not be detected in the present work. Steroid ketones were only found in minor amounts (30 mg/kg) in the respective rice husks waste (Marques et al., 2020), as well as in the related wheat straw, where they amounted to only 88 mg/kg (del Río et al., 2013a).

Finally, small amounts of steroid hydrocarbons (60 mg/kg) were also identified, including stigmasta-3,5-diene (20 mg/kg) and stigmasta-3,5,22-triene (**30**, 40 mg/kg). These compounds are most likely degradation products arising from free and conjugated sterols, as previously indicated (Marques et al., 2020).

The occurrence of important amounts of steroid compounds in rice straw makes it an interesting and useful raw material for obtaining valuable compounds of interest to the nutraceutical, pharmaceutical, cosmetic, food, and other industries. In particular, phytosterols are well known for their nutraceutical and health-promoting benefits, thus helping to reduce blood cholesterol levels, regulating cardiovascular disease, as well as exhibiting anticancer properties (Wilson et al., 2000; Moreau et al., 2002; Iwatsuki et al., 2003; Quílez et al., 2003; Jones and AbuMweis, 2009; Awika, 2011). However, on the other hand, as in the case of using rice straw as raw material for pulp and papermaking, the important amounts of steroid compounds, particularly free and conjugated (esterified and glycosylated) sterols, present in this material can be problematic as these compounds contribute significantly to the pitch deposits (del Río et al., 1998, 2000; Gutiérrez et al., 2001, 2004; Gutiérrez and del Río, 2001).

## Conclusion

The detailed composition of the lipophilic compounds in rice straw has been reported. The main compounds identified were *n*-fatty acids, *n*-alkanes, tocopherols, steroid hydrocarbons, steroid ketones, free sterols, sterol esters, sterol glucosides, mono-, di-, and triglycerides, and high molecular weight ester waxes. The data indicated that the amounts of most valuable phytochemicals are higher in rice straw than in the respective rice husks wastes or in other related cereal wastes such as in wheat straw. The composition of the lipophilic extractives reported here for rice straw, however, may vary depending on various factors (including cultivar., geographical location, age of crops, soil type, or climatic conditions, among others). Nevertheless, this information is of high interest for the valorization of rice straw as the lipophilic compounds can be obtained as a side-stream in a lignocellulosic biorefinery. These lipophilic compounds have a wide range of industrial applications and can be used for the nutraceutical, pharmaceutical, cosmetic, and chemical industries. In particularly, steroid compounds are of great interest in the pharmaceutical and nutraceutical as they have multiple health benefits, such as lowering plasma cholesterol levels, and they can be used as food supplements. However, for certain uses of rice straw, such as for pulp and papermaking, lipophilic compounds, and particularly the important amounts of free and conjugated sterols, represent a major problem because they are at the origin of the pitch deposits.

## Data Availability Statement

The raw data supporting the conclusions of this article will be made available by the authors, without undue reservation.

## Author Contributions

MJR and GM made the experimental work. AG and JR contributed to method development. JCR designed the work, processed the data, and wrote the article, with contributions from the rests of authors. All authors contributed to the article and approved the submitted version.

## Funding

This work was supported by the projects AGL2017-83036-R and PID2020-118968RB-I00 (funded by MCIN/AEI/10.13039/501100011033 and, as appropriate, by “ERDF A way of making Europe”) and the Regional Andalusian Government, Consejería de Transformación Económica, Industria, Conocimiento y Universidades/FEDER (project P20-00017). MJR acknowledges the Spanish Ministry of Science, Innovation and Universities for a FPI fellowship (PRE2018-083267).

## Conflict of Interest

The authors declare that the research was conducted in the absence of any commercial or financial relationships that could be construed as a potential conflict of interest.

## Publisher’s Note

All claims expressed in this article are solely those of the authors and do not necessarily represent those of their affiliated organizations, or those of the publisher, the editors and the reviewers. Any product that may be evaluated in this article, or claim that may be made by its manufacturer, is not guaranteed or endorsed by the publisher.
